# Modeling of pneumococcal serogroup 10 capsular polysaccharide molecular conformations provides insight into epitopes and observed cross-reactivity

**DOI:** 10.3389/fmolb.2022.961532

**Published:** 2022-08-08

**Authors:** Nicole I. Richardson, Michelle M. Kuttel, Neil Ravenscroft

**Affiliations:** ^1^ Department of Chemistry, University of Cape Town, Cape Town, South Africa; ^2^ Department of Computer Science, University of Cape Town, Cape Town, South Africa

**Keywords:** *S. pneumoniae*, serogroup 10, capsular polysaccharide, vaccine antigen, molecular modeling, cross-protection, carbohydrate epitopes

## Abstract

*Streptococcus pneumoniae* is an encapsulated gram-negative bacterium and a significant human pathogen*.* The capsular polysaccharide (CPS) is essential for virulence and a target antigen for vaccines. Although widespread introduction of pneumococcal conjugate vaccines (PCVs) has significantly reduced disease, the prevalence of non-vaccine serotypes has increased. On the basis of the CPS, *S. pneumoniae* serogroup 10 comprises four main serotypes 10A, 10B, 10C, and 10F; as well as the recently identified 10D. As it is the most prevalent, serotype 10A CPS has been included as a vaccine antigen in the next generation PCVs. Here we use molecular modeling to provide conformational rationales for the complex cross-reactivity reported between serotypes 10A, 10B, 10C, and 10F anti-sera. Although the highly mobile phosphodiester linkages produce very flexible CPS, shorter segments are conformationally defined, with exposed β -D-galactofuranose ( β DGal*f*) side chains that are potential antibody binding sites. We identify four distinct conformational epitopes for the immunodominant β DGal*f* that assist in rationalizing the complex asymmetric cross-reactivity relationships. In particular, we find that strongly cross-reactive serotypes share common epitopes. Further, we show that human intelectin-1 has the potential to bind the exposed exocyclic 1,2-diol of the terminal β DGal*f* in each serotype; the relative accessibility of three- or six-linked β DGal*f* may play a role in the strength of the innate immune response and hence serotype disease prevalence. In conclusion, our modeling study and relevant serological studies support the inclusion of serotype 10A in a vaccine to best protect against serogroup 10 disease.

## 1 Introduction

*S. pneumoniae* is an encapsulated gram-negative bacterium and an important human pathogen responsible for significant disease and mortality, especially in children under five (Geno et al., 2015). The capsular polysaccharide (CPS) is essential for virulence and an important target antigen for vaccines (Moreau and Schulz, 2000; Geno et al., 2015). Although 100 different serotypes have been identified, most disease is caused by a subset of pathogenic serotypes (Ganaie et al., 2020). Vaccines against the CPS of *S. pneumoniae* have been developed either as a pneumococcal polysaccharide vaccine (PPV) or a pneumococcal conjugate vaccine (PCV)—polysaccharide conjugated to a carrier protein (Geno et al., 2015). Unlike polysaccharide vaccines, the glycoconjugate vaccines induce a T cell dependent immune response and are effective in young children; the age group highly susceptible to infections (Lindberg, 1999). This has led to the licensure of PCVs targeting, initially, seven (PCV7, serotypes 4, 6B, 9 V, 14, 18C, 19F, and 23F), then ten (PCV10, PCV7 plus serotypes 1, 5, and 7F), and 13 (PCV13, PCV10 plus serotypes 3, 6A, and 19A) serotypes.

Widespread introduction of PCVs has significantly reduced the burden of invasive pneumococcal disease (IPD) due to vaccine serotypes (Wahl et al., 2018). However, increased prevalence of non-vaccine serotypes (due to serotype replacement as well as different geographical and socio-economic vaccination programs and prevalence of serotypes) means that IPD is still a leading cause of lower respiratory infection morbidity and mortality (GBD 2016 Lower Respiratory Infections Collaborators, 2018; Balsells et al., 2017; Wasserman et al., 2021). This necessitated the development of the third generation of PCVs, with higher serotype valency and protection against the emerging serotypes (Klugman and Rodgers, 2021). A PCV15 vaccine (PCV13 plus serotypes 22F and 33F) and a PCV20 vaccine (PCV15 plus serotypes 8, 10A, 11A, 12F, and 15B) have been licensed and higher valency vaccines such PCV24 (PCV20 plus serotypes 2, 9N, 17F, and 20) and PCV30 are in development (Klugman and Rodgers, 2021; Janssens et al., 2022; Kobayashi, 2022).

The epidemiology of serogroup 10 varies quite significantly across different geographical regions, with Pn10A considered the most prevalent, followed by Pn10B, with Pn10C and Pn10F occasionally isolated but not considered a major burden of disease (Balsells et al., 2017; dos Santos et al., 2013; Jauneikaite et al., 2012; Messaoudi et al., 2016; Wouters et al., 2019). The prevalent serotype 10A is present in PPV23 and targeted for inclusion in the third generation PCVs: in PCV20 and PCV24 (Jones, 1995; Yang et al., 2011; McGuinness et al., 2021; Janssens et al., 2022).

**FIGURE 1:**
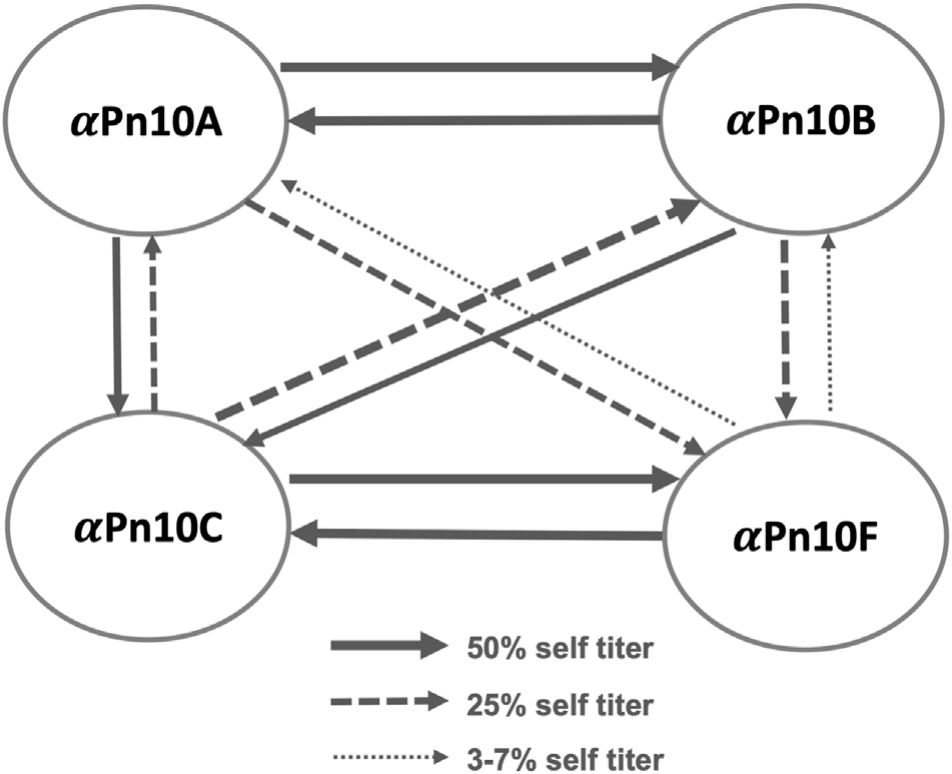
*S. pneumoniae* serogroup 10 rabbit antisera cross-reactivity trends showing the cross-reactivity of antisera ( α ) raised against serogroup 10 CPSs ( α Pn10A, α Pn10B, α Pn10C, and α Pn10F) (Henrichsen, 1995). Self-cross-reactivity titer was set as 100% with 50% self-titer, 25% self-titer, and 3%–7% self-titer represented by solid arrows, dashed arrows, and dotted arrows, respectively.

The literature reports considerable cross-reactivity within *S. pneumoniae* serogroup 10: between serotypes 10A (Pn10A), 10B (Pn10B), 10C (Pn10C), and 10F (Pn10F), summarized in Figure 1 (Henrichsen, 1995; Yang et al., 2011). Specifically, serotype-specific rabbit antisera ( α Pn) raised against one serotype recognized the CPS of the other serotypes, albeit with a lower affinity (Henrichsen, 1995). These data show some puzzling asymmetry, as follows. Strong reciprocal cross-reactivity was observed between Pn10A and Pn10B; as well as between Pn10C and Pn10F. Then, while Pn10A and Pn10B show strong cross-reactivity with Pn10C, the reciprocal cross-reactivity of Pn10C with these serotypes is weaker. Similarly, while Pn10A and Pn10B show moderate cross-reactivity with Pn10F, the reciprocal cross-reactivity of Pn10F is even weaker. The reasons for this asymmetry are unclear. Little information on the cross-reactivity and prevalence of the recently identified serotype 10D (Pn10D) is available (Ganaie et al., 2020).

**FIGURE 2:**
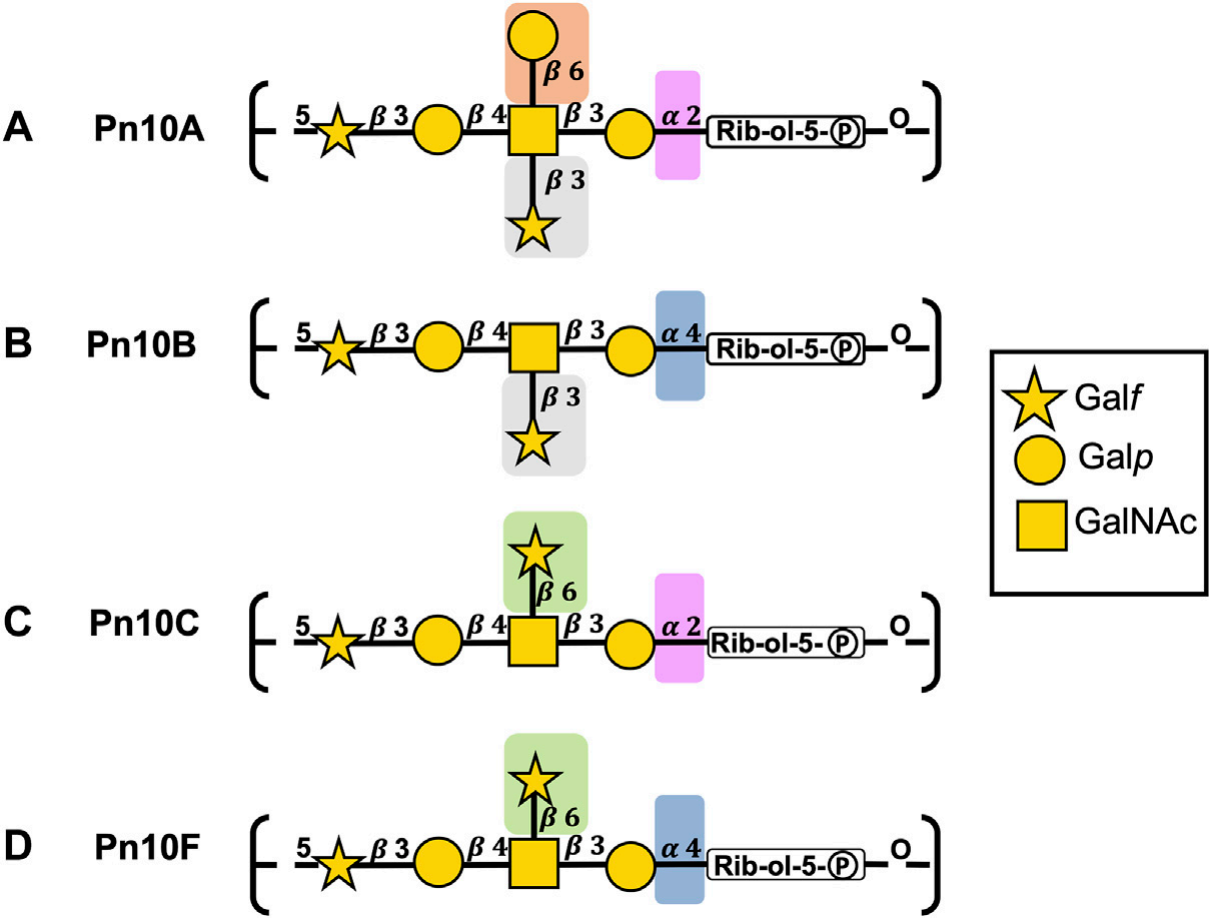
*S. pneumoniae* serogroup 10 CPS repeating unit structures for **(A)** Pn10A, **(B)** Pn10B, **(C)** Pn10C, and **(D)** Pn10F represented with the SNFG (Symbol Nomenclature for Glycans) system (Varki et al., 2015). Similarities and differences in repeating units are indicated by background color shading.

The repeating unit (RU) structures of the four main capsular polysaccharides in serogroup 10 (Table 1; Figure 2) contain the sugars β -D-galactofuranose ( β DGal*f*), β -D-galactopyranose ( β DGal), N-acetyl- β -D-galactosamine ( β DGalNAc), α -D-galactopyranose ( α DGal), and D-ribitol-5-phosphate (Rib-ol-5P), (Yang et al., 2010; Yang et al., 2011; Geno et al., 2015). Pn10A and Pn10C share a common backbone as do Pn10B and Pn10F; side chain substitutions create four structurally distinct CPSs. Note that Pn10D has a considerably different backbone to the rest of serogroup 10 (Ganaie et al., 2020) and so was not included in this study.

**TABLE 1:** Line structures of *S. pneumoniae* repeating units for serogroup 10 CPSs, side groups in bold.

	
Pn10A	[→5) β DGal*f* (1→3) β DGal*p* (1→4)**[** β **DGal*f* (1→3)][** β **DGal*p* (1→6)]** β DGal*p*NAc (1→3) α DGal*p* (1→2)DRib-ol-5P(P→]
Pn10B	[→5) β DGal*f* (1→3) β DGal*p* (1→4)**[** β **DGal*f* (1→3)]** β DGal*p*NAc (1→3) α DGal*p* (1→4)DRib-ol-5P(P→]
Pn10C	[→5) β DGal*f* (1→3) β DGal*p* (1→4)**[** β **DGal*f* (1→6)]** β DGal*p*NAc (1→3) α DGal*p* (1→2)DRib-ol-5P(P→]
Pn10F	[→5) β DGal*f* (1→3) β DGal*p* (1→4)**[** β **DGal*f* (1→6)]** β DGal*p*NAc (1→3) α DGal*p* (1→4)DRib-ol-5P(P→]
Pn10D	[→6) α DGlc*p* (1→3) α DGlc*p* (1→4)**[** β **DGal*f* (1→3)][** β **DGal*p* (1→6)]** β DGal*p*NAc (1→3) α DGal*p* (1→1)DRib-ol-5P(P→]

The backbones for Pn10A (Figure 2A) and Pn10C (Figure 2C) have a α DGal (1→2)Rib-ol-5P linkage Pn10B (Figure 2B) and Pn10F (Figure 2D) have a α DGal (1→4)Rib-ol-5P linkage. Furthermore, the substitutions on the branching β DGalNAc residue differ, as follows. Pn10B, Pn10C, and Pn10F are all singly substituted with β DGal*f*: at position three in Pn10B and at position six in both Pn10C and Pn10F. Pn10A has a double substitution on β DGalNAc: a β DGal*f* residue at position three and a β DGal residue at position six.

All serogroup 10 serotypes contain the β -D-galactofuranose monosaccharide— β DGal*f*. Absent in mammals, this sugar occurs in a range of bacterial pathogens (such as *Mycobacterium tuberculosis*, *Klebsiella pneumoniae* and *S. pneumoniae*) and is often critical for virulence and/or viability of the organism (Tefsen et al., 2012) and has been identified as an immunodominant epitope (Marino et al., 2017). Human intelectin-1 (hIntL-1) is a carbohydrate binding protein in the human innate immune system that recognizes the exocyclic terminal 1,2-diol (ETD) epitope which is present in galactofuranose, but not galactopyranose (Wesener et al., 2015; Kiessling, 2018; Isabella, 2021). This ETD motif is bound by hIntL-1 with high specificity in molecules such as D-*glycero*-D-*talo*-oct-2-ulosonic acid (KO), 3-deoxy-D-*manno*-oct-2-ulopyranosonic acid (KDO), β DGal*f*, and glycerol-1-phosphate (Kiessling, 2018; Isabella, 2021). As binding by hIntL-1 is associated with increased phagocytosis of the pathogens, pathogens have evolved to evade the immune system by modifying the ETD motif (such as masking with O-acetylation) thus preventing hIntL-1 recognition (Spencer et al., 2017; Chen et al., 2020).

Molecular modeling is a complementary technique that can be used to correlate the conformational features of carbohydrate antigens with data on serotype cross-protection produced by immunological studies (such as serological evaluations, animal trials, human trials, or epidemiological studies) (Zaccheus et al., 2012; Sarkar et al., 2013; Kang et al., 2014; Yang et al., 2017; Kuttel and Ravenscroft, 2018; Aytenfisu et al., 2019). Recently, we have explored the importance of the presentation of structural features or epitopes on bacterial CPS for serotype cross-protection (Richardson et al., 2021a; Kuttel, 2022). Here we apply our established molecular modeling methodology to the four primary *S. pneumoniae* serogroup 10 CPSs (Pn10A, Pn10B, Pn10C, and Pn10F) to establish their conformation and identify potential cross-protective epitopes. We aim to rationalize the observed cross-reactivity data and thus inform the design of the next generation of vaccines.

## 2 Materials and methods

We used our established systematic approach to the modeling of polysaccharides, as previously described (Hlozek et al., 2020; Richardson et al., 2021b), to build three repeating unit (3 RU) chains followed by 6 RU chains of Pn10A, Pn10B, Pn10C, and Pn10F in aqueous solution for initial molecular dynamics (MD) simulations.

Chain length is an important consideration when modeling CPSs, as a short chain may have insufficient molecular flexibility, while long chains are more computationally expensive to model. On the basis of our previous work, we consider a 6 RU chain to be representative of the behavior of the longer polysaccharide. Further, antibodies bind small fragments of the CPS between one and seven residues in length (Kabat, 1966) corresponding to 1 RU (or a fraction thereof) in the case of *S. pneumoniae* making a 6 RU model sufficient to explore antibody binding epitopes.

Following initial system equilibration, MD simulations were performed of 3 RU and 6 RU chains of each of the CPSs. Data analyses were performed on these production runs, as described below.

### 2.1 Molecular dynamics

The 3 RU and 6 RU chains were built using in-house CarbBuilder software (version 1.2.42) (Kuttel et al., 2011; Kuttel et al., 2016) and visualized with the Visual Molecular Dynamics (VMD) software (Humphrey et al., 1996). Starting structures for each molecule were built using low energy glycosidic linkages from potential mean force calculations, as per our previous work (Richardson et al., 2021b) and the psfgen tool was used by CarbBuilder to create protein structure (PSF) files for simulation with the CHARMM36 additive force field used to model the carbohydrates (Guvench et al., 2008; Guvench et al., 2009). The starting structures were subsequently minimized with the Nanoscale Molecular Dynamics (NAMD) program (version 2.13) for 10,000 steps at 300 K. Minimized structures were solvated using VMD’s built in solvation and ionization tools to add TIP3P (Jorgensen et al., 1983) cubic water boxes of 84 Å per side for 3 RU systems and 140 Å per side for 6 RU systems. Systems were then neutralized with one sodium (Na^+^) counter ion per repeating unit (three ions for the 3 RU, six for the 6 RU). Initial minimization and heating protocols comprised 5 K incremental temperature reassignments beginning at 10 K up to 300 K with 500 steps of NAMD minimization and 8,000 steps of MD at each temperature reassignment. Solvated and ionized structures (PDB and PSF files) for each 6 RU system are available as Supplementary Material.

Simulations of 3 RU were run using NAMD (version 2.13) (Phillips et al., 2005) with CUDA extensions for graphics processor acceleration (Stone et al., 2007); simulations of 6 RU were run using NAMD (version 3.0) with CUDA extensions for graphics processor acceleration and a GPU resident computational mode.

Periodic boundary conditions equivalent to the cubic box size were employed for the solvated simulation with wrapping on. Long range electrostatics were implemented with the Particle Mesh Ewald summation grid spacing set to 1 (Darden et al., 1993). Atoms were not held fixed, and the initial center of mass motion was off. The 1-3 pairs were excluded from non-bonded interactions, 1-4 interactions were not scaled, and the dielectric constant was set to 1. Smoothing functions were applied to both the electrostatics and van der Waals forces with switching and cut-off distances of 10 and 12 Å, respectively.

A Leap-Frog Verlet integrator was used to integrate the equations of motion over a step size of 1 fs. A distance of 15 Å was used as the cut-off for inclusion in the pair list for calculation of non-bonded forces. The short-range non-bonded interactions were calculated every 1 fs, full electrostatics calculations were performed every 2 fs, and atoms were reassigned every 10 fs (Van Gunsteren and Berendsen, 1988).

Simulations were sampled under isothermal-isobaric (nPT) ensemble. Langevin dynamics (Feller et al., 1995) were used to control the temperature with a damping coefficient of 5/ps. Nosé-Hoover Langevin piston dynamics were used as a barostat to maintain a target pressure of 1 atm (Nosé and Klein, 1983; Hoover, 1985). Variable system volume was used with a piston period of 100 fs and decay of 50 fs. Simulations of 3,000 ns were performed for the 6 RU systems (3 RU systems were run to 1,000 ns) comprising 200 ns of equilibration and 2,800 ns of production run as was required for convergence.

### 2.2 Convergence

We addressed convergence using block standard averaging (Grossfield and Zuckerman, 2009) applied to two metrics: end-to-end distance and radius of gyration (Supplementary Figure S1). Block standard averaging was implemented with in-house Python scripts.

For all simulations, the blocked standard error (BSE) reached plateaus for both metrics, indicating convergence. The simulation lengths were large multiples of the correlation times for end-to-end distance (Pn10A, 74 ns; Pn10B, 110 ns; Pn10C, 69 ns; Pn10F, 99 ns) and radius of gyration (Pn10A, 70 ns; Pn10B, 108 ns; Pn10C, 69 ns; Pn10F, 120 ns). Further, the numbers of independent samples were >>1 for both the end-to-end distance (Pn10A, 40; Pn10B, 27; Pn10C, 45; Pn10F, 30) and the radius of gyration (Pn10A, 43; Pn10B, 28; Pn10C, 44; Pn10F, 25). Our designated equilibration time of 200 ns is therefore greater than the correlation time.

### 2.3 Data analysis

Molecular conformations were visualized using VMD, with the PaperChain and Twister visualization algorithms used to highlight carbohydrate rings and chains (Cross et al., 2009) as required.

Trajectories were extracted at 25 ps intervals with analysis of performed on frames 250 ps apart. Metrics such as end-to-end distances were extracted from the simulation trajectories using Tcl scripting *via* VMD’s Tk console. Data analyses performed with in-house Python scripts and plots generated using Matplotlib (Hunter, 2007).

#### 2.3.1 Chain flexibility

The end-to-end distance, *r*, was measured from C1 of α DGal at the non-reducing end, to C1 of β DGal at the reducing end thus excluding the highly flexible terminal residues.

#### 2.3.2 Conformational analysis

The most common chain conformations for each simulation were determined by clustering the production trajectory frames into families and calculating the relative occupancies of each family. Clusters comprising less than 6% of the production run (post equilibration) were discarded. Clustering was performed using the WMC PhysBio plug-in for VMD’s built-in measure cluster command (Gracia Luis, 2012). Prior to clustering, the molecules were aligned on the RU 4 backbone excluding the Rib-ol-5P residue and any hydrogens on the backbone. Clustering was then performed as an RMSD fit to the ring and linkage atoms of the central four repeating units of the chains, excluding the highly flexible terminal RU 1 and RU 6. Eight clusters were created with a cut-off of 8 Å.

Clustering analysis was also performed on a single repeating unit, RU 4, and on the β DGalNAc— β DGal*f* linkage of RU 4. For the RU 4 analysis, molecules were aligned on the β DGalNAc residue of RU 4, excluding hydrogens. Clustering was then performed on the ring and linkage atoms of RU 4, creating five clusters with a cutoff of 1.5 Å, before discarding clusters comprising less than 10% of the trajectory.

Similarly, for the β DGalNAc— β DGal*f* linkage, molecules were aligned on the β DGalNAc residue of RU 4 and clustering was then performed on the β DGalNAc and β DGal*f* ring and linkage atoms. Four clusters were created with a cut-off of 1.5 Å and clusters comprising less than 6% of the production run (post-equilibration) were discarded.

### 2.4 hIntL-1 binding

The protein data bank (PDB) file describing the structure of human intelectin-1 (hIntL-1) bound to allyl- β -galactofuranose (allyl β Gal*f*) (PDB ID: 4WMY) was obtained from the official RSCB PDB (Wesener et al., 2015). Overlay of our CPS molecules with the hIntL-1 protein binding site were created by aligning the O4, C5, O5, C6, and O6 atoms of the central side group β DGal*f* residue from each CPS molecules with that of the allyl β DGal*f* in the hIntL-1 binding site. We then identified frames where the molecular conformation aligns with the hIntL-1 binding site free from protein-CPS intersection with good alignment of the galactofuranose rings and exocyclic terminal-1,2-diol (ETD) moieties. For the 3 RU molecules, we aligned β DGal*f* from RU 2 and for the 6RU molecules, β DGal*f* from RU 3.

## 3 Results

We begin with a comparison of the flexibility of the serogroup 10 CPSs. This is followed by analysis of the CPS molecular conformation, exposed potential binding epitopes and, finally, an exploration of the potential for CPS binding to the hIntL-1 human innate immune protein.

### 3.1 Chain flexibility and conformation

**FIGURE 3:**
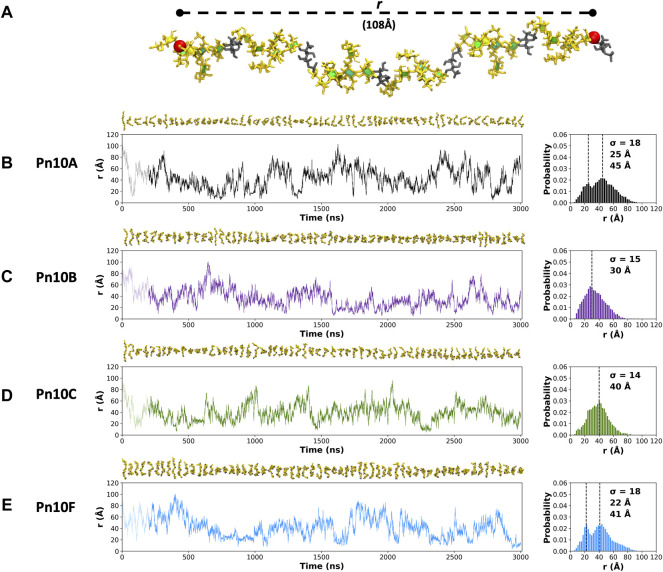
**(A)** The end-to-end distance, *r*, is indicated on the 6 RU Pn10A molecule. Time series graphs (left column) of *r* and corresponding histograms (right column) for the 3,000 ns simulation trajectories are shown for: **(B)** Pn10A, **(C)** Pn10B, **(D)** Pn10C, and **(E)** Pn10F. Conformational snapshots at 50 ns intervals are shown above the time series plots. The histograms are labeled with the standard deviations ( σ ) and modal peak *r* value(s).

The fluctuation in molecular end-to-end distance, *r*, over the length of the simulation is commonly used as a measure of chain extension and flexibility in carbohydrates. Here we define *r* to exclude the mobile terminal residues (Figure 3A). Times series plots of *r* for each CPS (Figures 3B–E, left column) reveal that all four molecules are highly flexible, fluctuating between a wide range of *r* values. Trajectory snapshots at 50 ns intervals (shown above the graphs) illustrate the considerable diversity in the molecular conformations over all the simulations. This flexibility is an expected consequence of a linear alditol (Rib-ol-5P) with a phosphodiester linkage in the CPS backbone: modeling of *Haemophilus influenzae* serotypes a and b CPS, which also contain this moiety, showed similarly flexible molecules (Richardson et al., 2021a).

Comparison of the *r* distributions (Figures 3B–E, right column) reveals some broad differences between the serotypes: Pn10A and Pn10F have bimodal distributions, whereas Pn10B and Pn10C are closer to unimodal, narrower and somewhat shifted to smaller values of *r*. Interestingly, it is the most structurally dissimilar pairs of molecules—Pn10A and Pn10F (Figures 2B,E); Pn10B and Pn10C (Figures 2C,D)—that are most similar with respect to the *r* distributions. While all four molecules are flexible, these data suggest an order of flexibility of Pn10A ∼ Pn10F > Pn10B > Pn10C and, relatively speaking, a slightly increased incidence of extended conformations for Pn10A and Pn10F.

**FIGURE 4:**
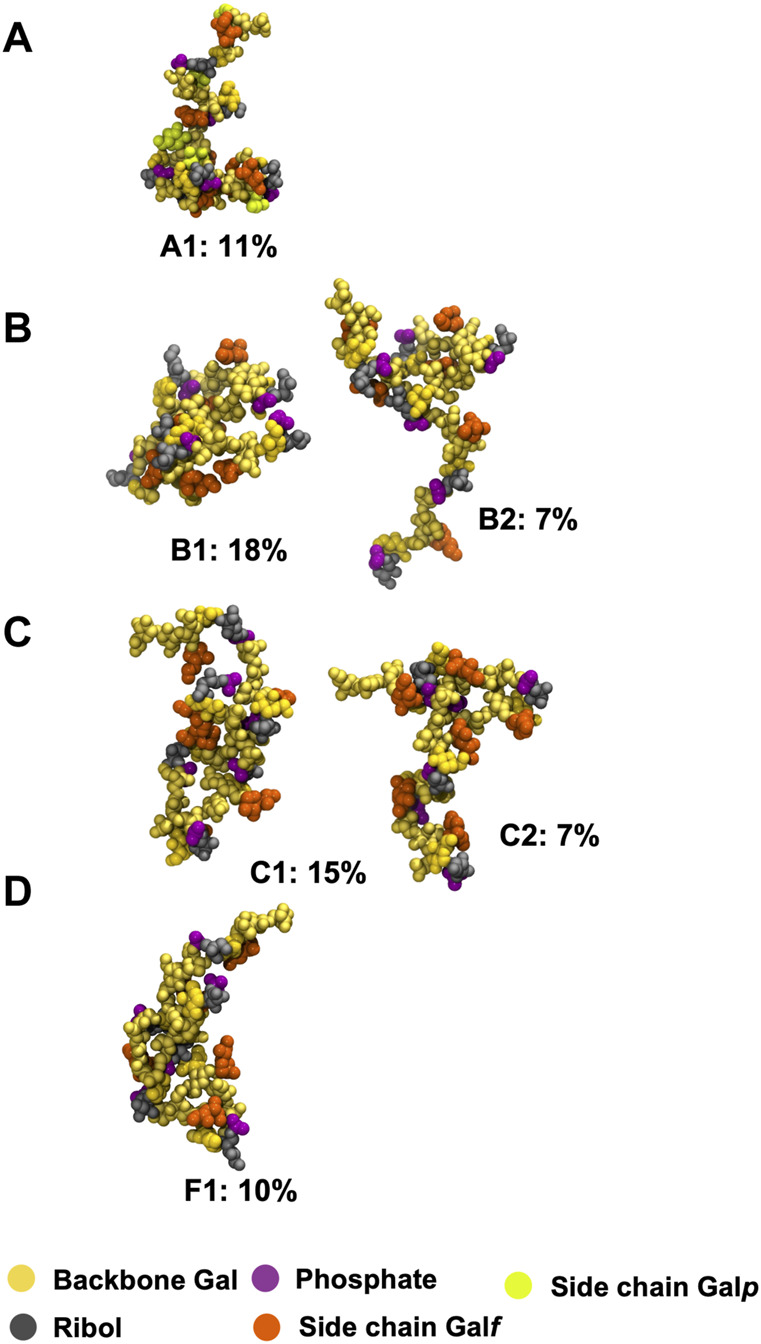
Main conformational families identified for the 6 RU *S. pneumoniae* serotype CPS, with the terminal repeat units (RU 1 and RU 6) excluded from analysis: **(A)** Pn10A, **(B)** Pn10B, **(C)** Pn10C, and **(D)** Pn10F. Backbone galactose residues are shown in yellow, ribitol residues shown in grey, phosphate in purple, side group galactofuranose residues in orange, and side group galactopyranose residues in light green. The primary clusters occurred regularly throughout the trajectory. The secondary cluster of Pn10B occurred regularly in the second half of the simulation along with the primary cluster; for Pn10C the secondary cluster occurred only for ∼400 ns around the 1,500 ns timestep.

The extreme flexibility of the CPS backbone means that there are no dominant chain conformations for any of the four carbohydrates (Figure 4). For RU 2 to RU 5 of the CPS backbone (i.e., 20 backbone residues) we found very few significant conformational families that occupy more than 6% of the simulation. Further, those we identified accounted for relatively small portions of the simulation (less than 25%). This indicates that these flexible chains behave as random coils, in common with other flexible carbohydrates that we have modeled (Richardson et al., 2021a; Richardson et al., 2021b).

We therefore conclude that none of these flexible CPS show evidence for a conformational epitope. However, most of the CPS chain flexibility originates from the backbone phosphodiester linkage to the Rib-ol-5P in the CPS backbone, with the other glycosidic linkages being relatively constrained (Supplementary Figure S2), potentially forming stable epitopes. Further, in all four molecules, the side chain β DGal*f* and β DGal residues are solvent exposed (Figure 4) and are therefore accessible for antibody binding. As antibodies bind to carbohydrate epitopes that typically comprise one to seven residues (Kabat, 1966), it proved more useful to compare the 6 RU CPS we modeled on this smaller length scale.

### 3.2 Epitopes

**FIGURE 5:**
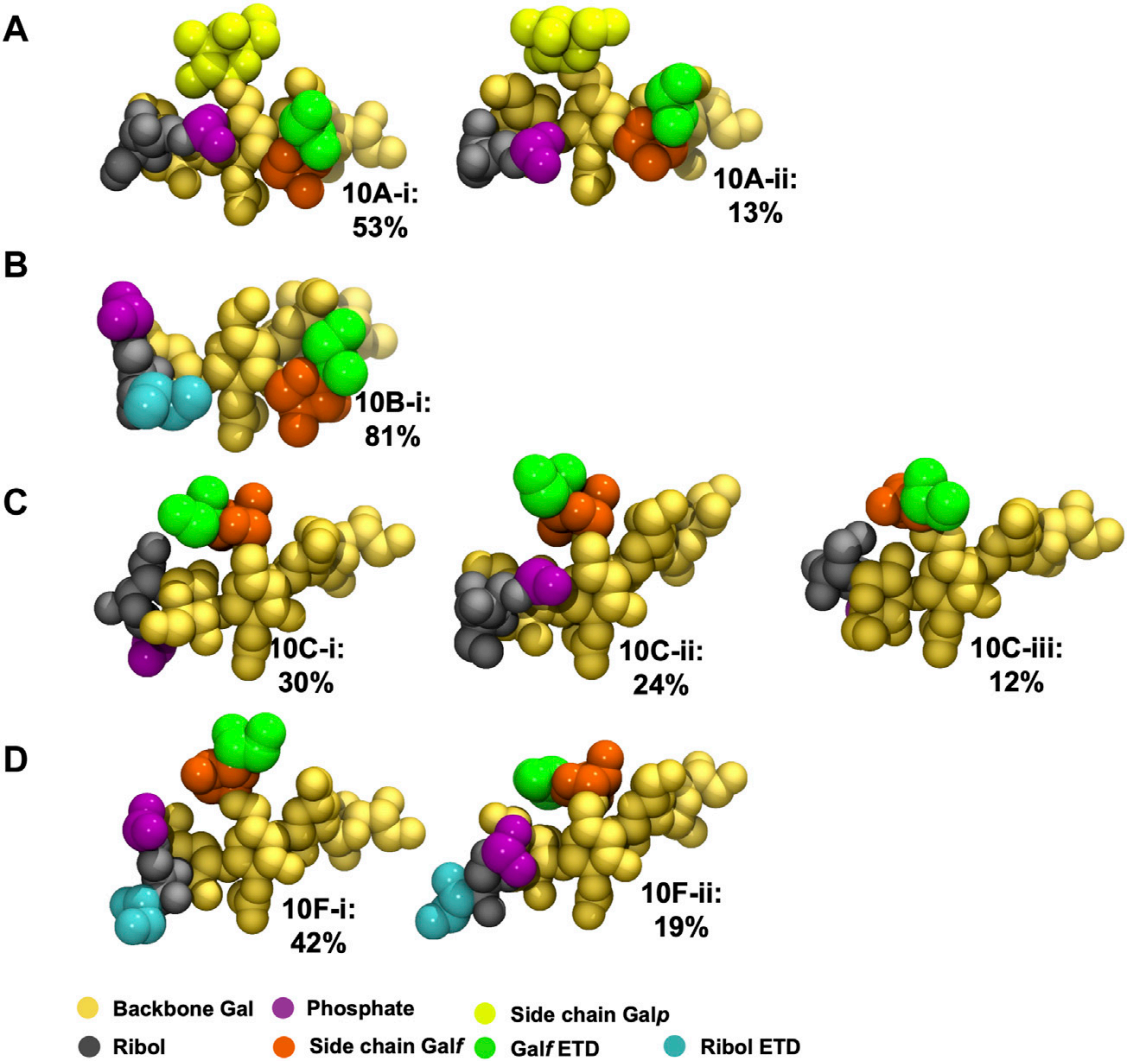
Main conformational families identified for RU 4 in the backbone of 6 RU *S. pneumoniae* serotype CPS: **(A)** Pn10A, **(B)** Pn10B, **(C)** Pn10C, and **(D)** Pn10F. Backbone galactose residues shown in yellow, Rib-ol-5P residues shown in grey, phosphate in purple, side group β Gal*f* residues in orange, side group β Gal residues in light green, ETD portion of β Gal*f* in green, and ETD portion of Rib-ol-5P residues in cyan.

The principal conformations for the single central repeating unit (RU 4) in each of the CPS chains are shown in Figure 5. With the highly mobile phosphodiester linkage excluded, we find dominant conformations with high occupancies ranging from 30% to 80% for the primary cluster and 13%–24% for the secondary cluster; more than 50% of the simulation trajectory falls within these dominant clusters. For Pn10A (Figure 5A) the 6-linked β DGal side chain (yellow) protrudes from the backbone and is located close to the phosphate group, providing a conformational rational for the dominance of β DGal as an antibody binding epitope for Pn10A (Nahm et al., 2020). In contrast, the immunodominant β DGal*f* side chain in Pn10A is 3-linked and aligned with the backbone and thus less exposed for binding. This is also the case for the 3-linked β DGal*f* side chain in Pn10B (Figure 5B), which is in a similar orientation to Pn10A. However, Pn10C and Pn10F (Figures 5C,D) have a 6-linked β DGal*f* side chain, which is considerably more exposed and thus a more promising epitope. This 6-linked side chain is also more flexible than the 3-linked β DGal*f* in Pn10A and Pn10B; it is oriented in different directions across the dominant conformational clusters in Pn10C and Pn10F. As the β DGal*f* residue is conserved across the serogroup, this immunodominant epitope could form the basis of the observed serotype cross-reactivity. In particular, for β DGal*f*, the exocyclic 1,2-terminal diol (ETD, colored green in Figure 5) is a potential epitope for CPS binding of the hIntL-1 human innate immune protein. A second ETD from the 4,5-linked ribitol of Rib-ol-5P is present in Pn10B and Pn10F, shown in cyan in Figures 5B,D.

**FIGURE 6:**
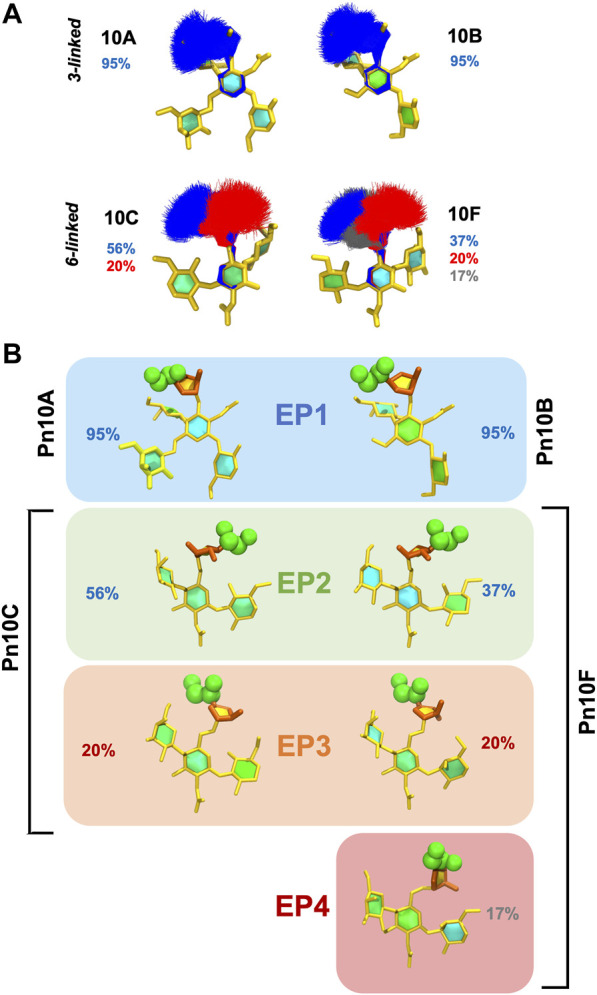
Conformational epitopes of the β DGalNAc— β DGal*f* disaccharide in *S. pneumoniae* serogroup 10, RU 4. **(A)** Super-imposed conformational families (>5%) of the β DGalNAc— β DGal*f* disaccharide with associated percentages for the four CPS molecules. **(B)** Representative conformational epitopes (EP1-4) of the β DGalNAc— β DGal*f* disaccharide for the four CPS molecules, with associated percentage occupancies, grouped according to conformational similarity.

To explore the range of conformations of the immunodominant β DGal*f* epitope in finer detail, we focus on the conformations of the β DGalNAc— β DGal*f* disaccharide in the CPS chain in Figure 6. The primary (blue) and secondary (red) conformational families are super-imposed in Figure 6A for all four serotypes. It is clear that 3-linked β DGal*f* (Pn10A and Pn10B) side chains have a single dominant conformation (>90% occupancy), whereas Pn10C and Pn10F, with 6-linked β DGal*f*, do not. Pn10C has two main conformations (56% and 20% occupancy, respectively) while Pn10F has three (37%, 20%, and 17% occupancy, respectively). Comparison of representative structures for each conformational family suggests four distinct conformational epitopes (EP) of the β DGalNAc— β DGal*f* disaccharide across the serotypes (Figure 6B). Pn10A and Pn10B share the same dominant conformational epitope of 3-linked β DGal*f*, which we term EP1. Pn10C and Pn10F also share a main conformation for their 6-linked β DGal*f*, which we term EP2, as well as a secondary conformation (EP3). Pn10F also has a unique conformation of this disaccharide, which we term EP4. Further—considering the β DGal*f* and ETD orientations relative to the backbone—EP1 and EP3 are similar while EP2 is related to EP3 by a 180° rotation; EP4 is a distinct epitope.

These conformational epitopes identified for β DGal*f* help to rationalize the trends in cross-reactivity observed for serogroup 10 (Figure 1), as follows. Firstly, the strong reciprocal cross-reactivity of Pn10A with Pn10B can be attributed to the shared EP1 epitope (Figure 6B, first panel). Similarly, the shared epitopes EP2 and EP3 explain the strong reciprocal cross-reactivity between Pn10C and Pn10F (Figure 6B, second and third panels). Then, the cross-reactivity of Pn10A/Pn10B with Pn10C/Pn10F can be explained by the conformational similarities between EP1 and EP2/EP3 (Figure 6B, first three panels). Further, for Pn10A/Pn10B the cross reactivity is stronger with Pn10C than with Pn10F, because the combined prevalence of the EP2 and EP3 epitopes is considerably more in Pn10C (76%) than in Pn10F (57%). Likewise, the weaker reciprocal cross-reactivity of Pn10C with these serotypes can be explained by the lower prevalence of the EP2 and EP3 conformational epitopes in Pn10C—fewer antibodies generated against these epitopes would hence, be less likely to recognize EP1 dominant in Pn10A/Pn10B. Similarly, the still weaker reciprocal cross-reactivity of Pn10F can be explained by the even lower expression of EP2 and EP3 as well as an additional conformational epitope, EP4, not shared by any of the other serotypes.

### 3.3 hIntL-1 binding

**FIGURE 7:**
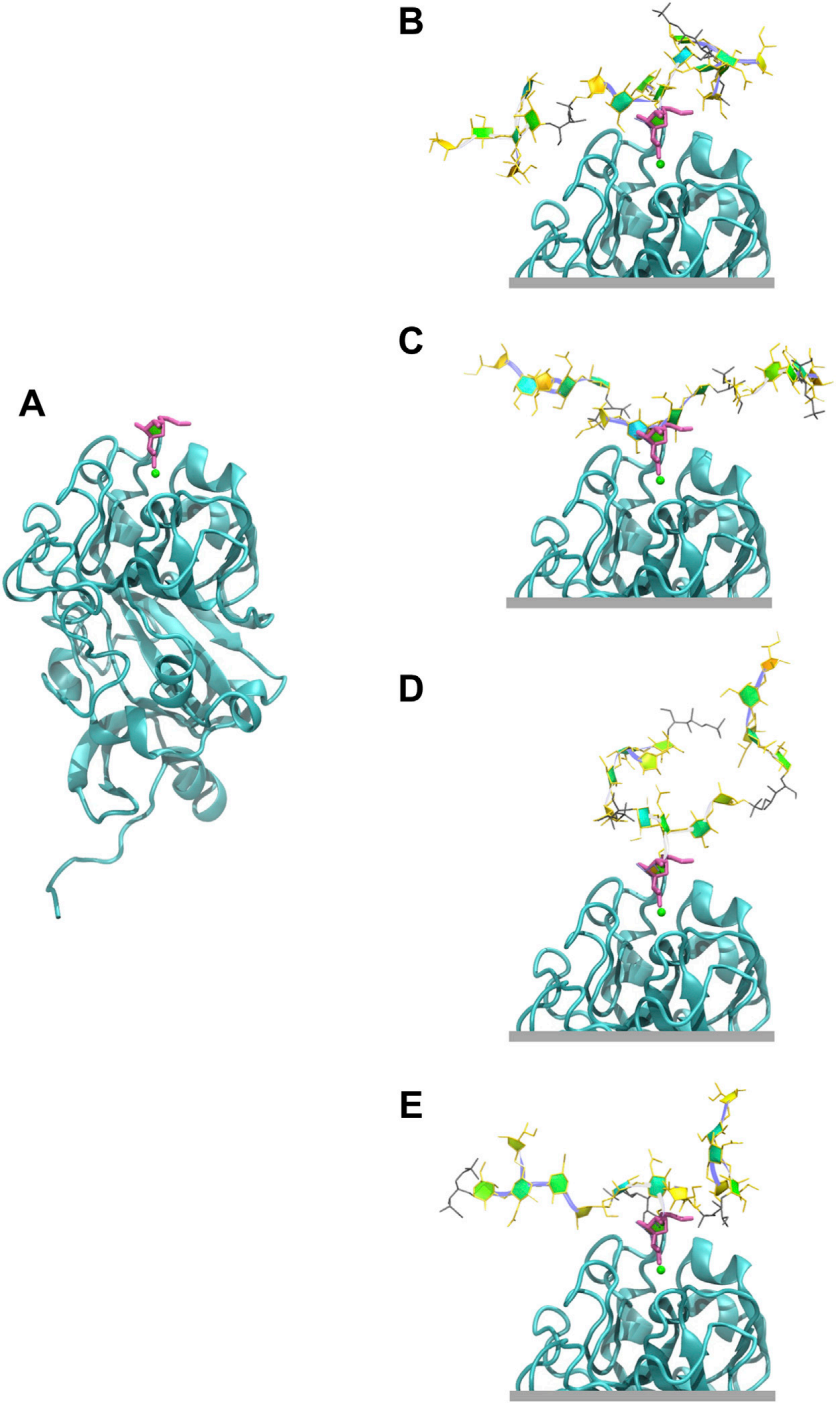
Human intelectin-1 (hIntL-1) binding. **(A)** Monomer of the crystal structure of hIntL-1 bound with allyl-beta-galactofuranose (PDB ID: 4WMY). Example conformations of the 3 RU CPSs with the β DGal*f* side chain positioned in the hIntL-1 binding site are shown for **(B)** Pn10A, **(C)** Pn10B, **(D)** Pn10C, and **(E)** Pn10F. The end-to-end distance (*r*) of these conformations are 33 Å, 41 Å, 16 Å, and 33 Å for Pn10A, Pn10B, Pn10C, and Pn10F, respectively. Overlay of our CPS molecules with the hIntL-1 protein binding site were created by aligning the O4, C5, O5, C6, and O6 atoms of the central side group (RU 2) β DGal*f* residue from each CPS molecule with that of the allyl β DGal*f* in the hIntL-1 binding site. We then identified frames where the molecular conformation aligns with the hIntL-1 binding site free from protein-CPS intersection with good alignment of the galactofuranose rings and exocyclic terminal-1,2-diol (ETD) moieties.

The exocyclic 1,2-terminal diol on β DGal*f* (ETD) is a potential epitope for CPS binding to the hIntL-1 human innate immune protein. We used the crystal structure of hIntL-1 bound to allyl β DGal*f* (Figure 7A)(Kiessling, 2018; Isabella, 2021) as a template for possible binding of the serogroup 10 CPS. Example conformations of the 3RU CPS with the central β DGal*f* ETD in the chain super-imposed on the ETD in the hIntL-1 binding site are shown in Figures 7B–E. These structures illustrate that it is possible for hIntL-1 to bind the EP1/EP2 epitope without involvement of the CPS backbone. However, the 3-linked β DGal*f* on Pn10A and Pn10B results in closer proximity of the CPS backbone to the protein than the 6-linked β DGal*f* on Pn10C and Pn10F, suggesting that these serotypes will bind less readily. For the 6 RU CPS chains we identified similar conformations (Supplementary Figure S3). Interestingly, the more collapsed conformations (Supplementary Figures S3A,B) have better potential for binding, as the β Gal*f* side chain projects from the backbone, with consequently less protein-CPS entanglement with the flexible backbone. Lastly, Supplementary Figure S4 presents the potential hIntL-1 binding of the ETD of Rib-ol-5P present in Pn10B and Pn10F.

## 4 Discussion

Our simulations show that *S. pneumoniae* serogroup 10 have highly flexible CPSs, which is unsurprisingly due to the backbone linear Rib-ol-5P alditol. Highly flexible bacterial CPSs are common and we have previously suggested that this is a strategy for bacterial evasion of the host immune system: a flexible backbone with multiple conformations presents a “moving target” to the immune system (Kuttel and Ravenscroft, 2019). For serogroup 10, this mobility may be especially necessary given the presence of an immunodominant PO_4_ group (Zhang et al., 2021) and the presence of a non-mammalian galactofuranose sugar to which the human immune system has a natural defense. Between the highly mobile phosphodiester linkages to Rib-ol-5P, the segments of the CPS chain are relatively rigid and the β DGal*p* and β DGal*f* side chains form exposed and well-defined epitopes. In particular, the β DGal*f* side chains are common to all four serotypes and considered to be immunodominant (Marino et al., 2017). Our analysis of the conformational epitopes of β DGal*f* provides a rationalization of the observed cross-reactivity between the serotypes in serogroup 10. We suggest that the varying flexibilities found for the four β DGal*f* conformational epitopes identified has relevance for their observed complex patterns of cross-reactivity, as follows. The relatively immobile 3-linked β DGal*f* forms the EP1 epitope in Pn10A and Pn10B; it is dominant (95%) in both serotypes and therefore there is strong cross-reactivity between them. This suggests cross-protection of a Pn10A vaccine against Pn10B, and also accounts for the reported mistyping of a Pn10A isolate as Pn10B (Konradsen, 2005).

The more flexible 6-linked β DGal*f*, present in both Pn10C and Pn10F, gives rise to the conformational epitopes EP2 and EP3, which are similar to the EP1 epitope and suggests some cross-protection for a Pn10A vaccine against both Pn10C and Pn10F. The relative proportions of EP2 and EP3 expressed in the serotypes accounts for the asymmetry of cross-reactivity (Figure 1), as the stronger cross-reactivity of Pn10A/Pn10B with Pn10C than with Pn10F corresponds to a higher prevalence of the EP2/EP3 epitopes in Pn10C (76% versus 57%). This is in line with our modeling of the CPS from meningococcal serogroups Y and W (Kuttel et al., 2017): we found that the less flexible serogroup Y CPS has a single dominant conformation, which corresponds to only one of several conformational epitopes exhibited by the closely related, but more flexible, serogroup W CPS. This explained the asymmetric cross-protection observed in the clinic: vaccination with serogroup Y elicited cross-protection against serogroup W in the majority of subjects, whereas minimal cross-protection was observed against serogroup Y following serogroup W vaccination. Further, the β DGal*f* EP1 epitope could correspond to the factor serum 10d epitope reported in the literature, as 10d reacts strongly with Pn10A and Pn10B (displaying EP1), but only weakly with Pn10C and Pn10F (displaying EP2/EP3) (Yu et al., 2005; Ganaie et al., 2020).

Pn10C and Pn10F also share both the β DGal*f* EP2 and EP3 conformational epitopes, which explains their strong mutual cross-reactivity, although Pn10F has an additional conformational epitope EP4 (17%) not present in the other serotypes.

The recently discovered serotype Pn10D has a different backbone (and was not modeled in this study), but also exposes the 6-linked β DGal*p* and 3-linked β DGal*f* side chains found in Pn10A (Ganaie et al., 2020). As expected, immunization with a polysaccharide vaccine containing Pn10A raised cross-opsonic antibodies against Pn10D, further confirming the inclusion of serotype 10A in a vaccine to best protect against serogroup 10 disease. These side chains are also present in *S. pneumoniae* serotype 39 which shows cross-reaction with factor serum 10d that reacts with Pn10A (Jones, 1995). Partial O-acetylation on the terminal β DGal*f* in serotype 39 may be an example of masking of this key epitope to escape innate immunity (Bush et al., 2014; Petersen et al., 2014).

Finally, we showed that the hIntL-1 human innate immune protein has the potential to bind the EP1/EP2 epitope in serogroup 10, containing the exocyclic 1,2-terminal diol on β DGal*f*. Binding of 3-linked β DGal*f* EP1 epitope in Pn10A and Pn10B results in closer proximity of the CPS backbone to the protein than for the more accessible 6-linked β DGal*f* EP2 epitope in Pn10C and Pn10F, suggesting that serotypes Pn10A/Pn10B will be bound less readily. This may play a role in the strength of the innate immune response and hence serotype disease prevalence with more cases reported for Pn10A/Pn10B than Pn10C/Pn10F.

In conclusion, the common epitopes we have identified for serogroup 10 support a vaccine containing serotype 10A providing adequate cross-protection against serogroup 10 disease, as indicated by serological studies.

## Data Availability

The datasets presented in this study can be found in online repositories. The names of the repository/repositories and accession number(s) can be found below: https://www.rcsb.org/structure/4WMY.

## Glossary

α DGal: α -D-galactopyranose
α DGlcp: α -D-glucopyranose
allyl β Galf: allyl- β -galactofuranose
α Pn: serotype-specific rabbit antisera
β DGal: β -D-galactopyranose
β DGalf: β -D-galactofuranose
β GalNAc: N-acetyl- β -D-galactosamine
**BSE**: block standard averaging
**CPS**: capsular polysaccharide
**CUDA**: compute unified device architecture
**EP**: epitope
**ETD**: exocyclic terminal-1,2-diol
**GPU**: graphics processing unit
**hIntL-1**: human intelectin-1
**IPD**: invasive pneumococcal disease
**KDO**: 3-deoxy-D-manno-oct-2-ulopyranosonic acid
**KO**: D-glycero-D-talo-oct-2-ulosonic acid
**MD**: molecular dynamics
**NAMD**: nanoscale molecular dynamics
**nPT**: isothermal-isobaric
**PCV**: pneumococcal conjugate vaccine
**PDB**: Protein Data Bank
**Pn10A**: *S. pneumoniae* serotype 10A
**Pn10B**: *S. pneumoniae* serotype 10B
**Pn10C**: *S. pneumoniae* serotype 10C
**Pn10D**: *S. pneumoniae* serotype 10D
**Pn10F**: *S. pneumoniae* serotype 10F
**PPV**: pneumococcal polysaccharide vaccine
**PSF**: protein structure file
**r**: end-to-end distance
**Rib-ol-5P**: D-ribitol-5-phosphate
**RMSD**: root-mean-square deviation
**RSCB**: Royal Society of Chemistry Biology
**RU**: repeating unit
**VMD**: visual molecular dynamics
