# Supplementary material for: Modeling of pneumococcal serogroup 10 capsular polysaccharide molecular conformations provides insight into epitopes and observed cross-reactivity
Source: Front Mol Biosci. 2022 Aug 8;9:961532. doi: 10.3389/fmolb.2022.961532 (PMC9393222; doi:10.3389/fmolb.2022.961532)
Supplement: Supplementary file 1 [file Table1.DOCX]

Supplementary Material

## Supplementary Figures


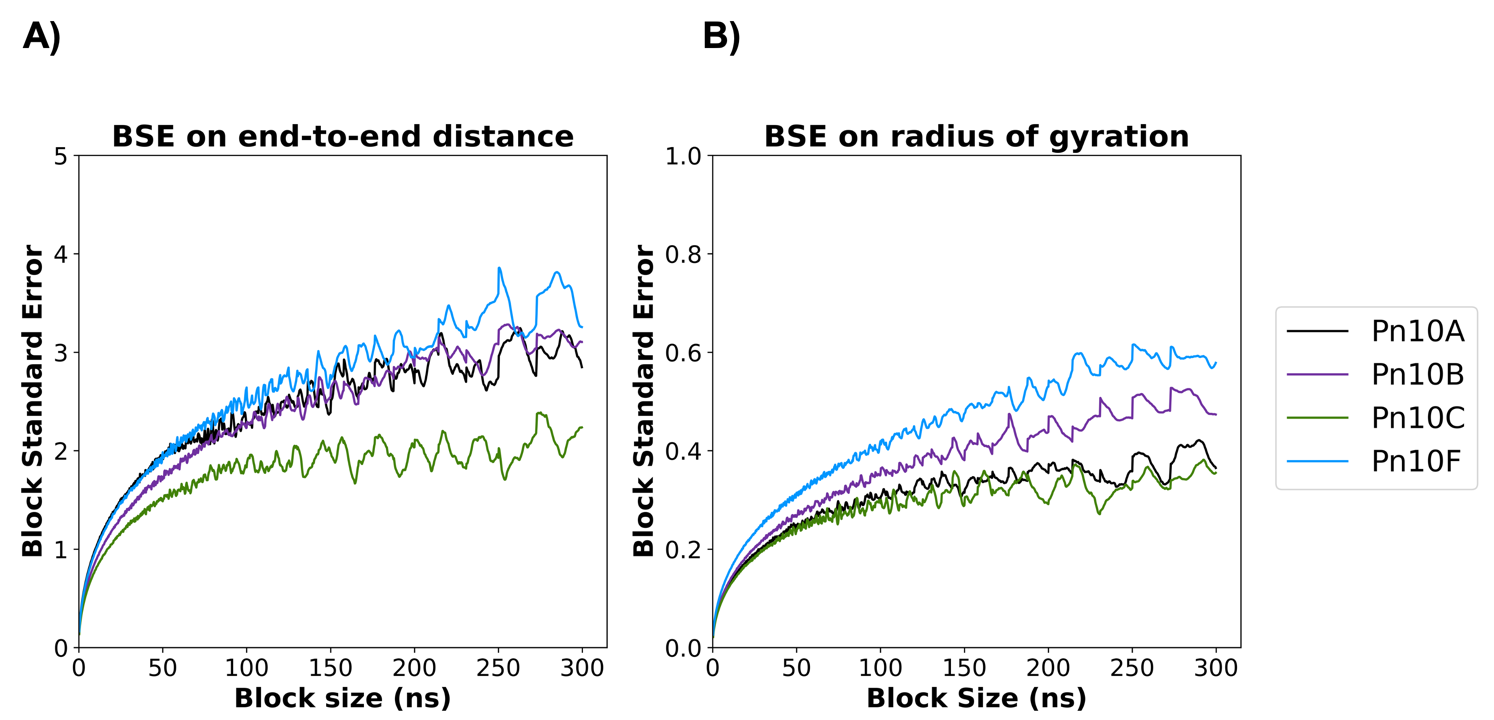


**Supplementary Figure 1.** Block standard averaging analysis for modeled *S. pneumoniae* serogroup 10 CPS molecules. **(A)** block standard error (BSE) versus block size (ns) calculated on end-to-end distance. **(B)** BSE versus block size calculated on radius of gyration. For all molecules, the BSE visually reaches a plateau.


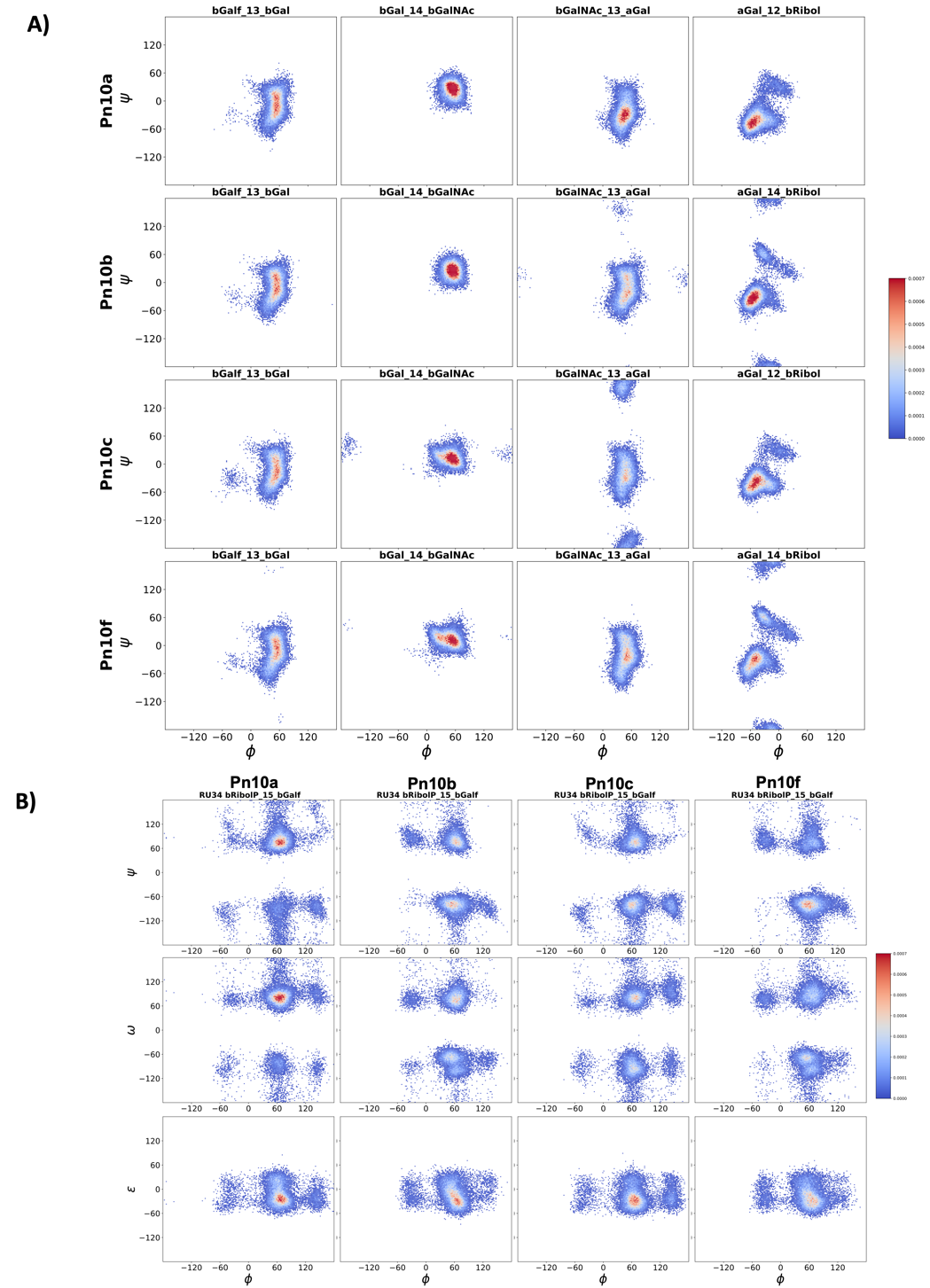


**Supplementary Figure 2.** Heatmap plots of dihedral angles for backbone linkages. **(A)** glycosidic linkages phi ($\phi$) vs psi ($\psi$) for the $\beta$DGal*f* – $\beta$DGal; $\beta$DGal – $\beta$DGalNAc; $\beta$DGalNAc – $\alpha$DGal; and $\alpha$DGal – $\beta$DRib-ol-5P linkages for each serotype. **(B)** phosphodiester linkages $\phi$ vs $\psi$, omega ($\omega$), and epsilon ($\varepsilon$) for the $\beta$DRib-ol-5P – $\beta$Gal*f* linkage of each serotype. The phosphodiester linkages are more flexible than the glycosidic linkages. The linkages were defined as we have done previously for these types of linkages (Richardson et al., 2021). Glycosidic linkages were defined as $\phi$ = H1-C1-O1-Cx’ and $\psi$ = C1-O1-Cx’-Hx’ and the phosphodiester linkages were defined as: $\phi$ = Hx-Cx-Ox-P, $\psi$ = Cx-Ox-P-Oy, $\omega$ = Ox-P-Oy-Cy and $\varepsilon$ = P-Oy-Cy-Hy.


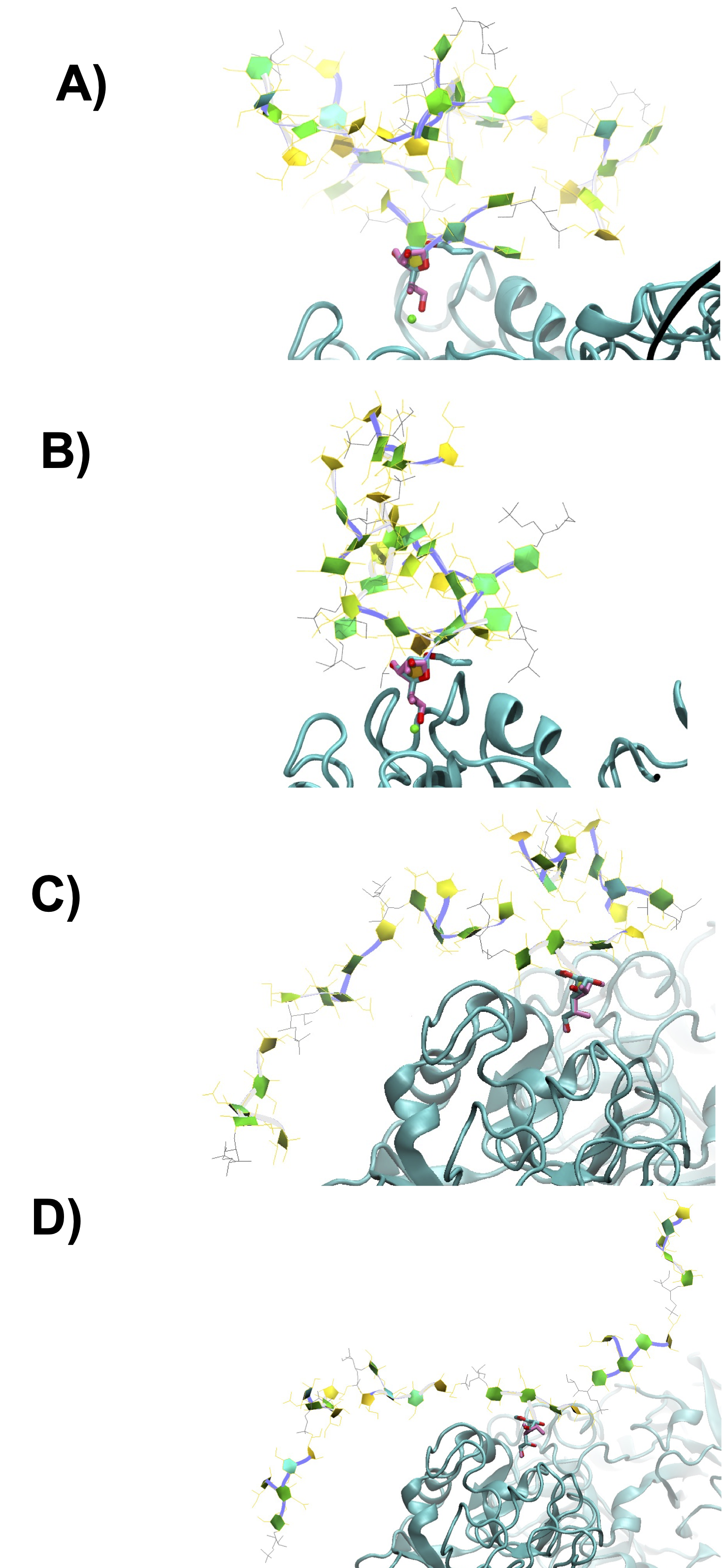


**Supplementary Figure 3.** Alignment of human intelectin-1 bound to allyl-$\beta$-galactofuranose (hIntL-1) with *S. pneumoniae* serogroup 10 CPS $\beta$DGal*f* ETD moieties. Alignment of 6 RU CPSs RU 4 side group $\beta$DGal*f* with hIntL-1 binding site for: **(A)** Pn10A, **(B)** Pn10B, **(C)** Pn10C, and **(D)** Pn10F. All four molecules align well and would be expected to bind. It was noted that collapsed chain conformations as in **(A)** and **(B)** were easier to align as there was less CPS-protein intersection. The protein data bank (PDB) file describing the structure of human intelectin-1 (hIntL-1) bound to allyl-$\beta$-galactofuranose (allyl $\beta$Gal*f*) (PDB ID: 4WMY) was obtained from the official RSCB PDB (Wesener et al., 2015). Overlay of our CPS molecules with the hIntL-1 protein binding site were created by aligning the O4, C5, O5, C6, and O6 atoms of the central side group (RU 3) $\beta$DGal*f* residue from each CPS molecule with that of the allyl $\beta$DGal*f* in the hIntL-1 binding site. We then identified frames where the molecular conformation aligns with the hIntL‑1 binding site free from protein-CPS intersection with good alignment of the galactofuranose rings and exocyclic terminal-1,2-diol (ETD) moieties.


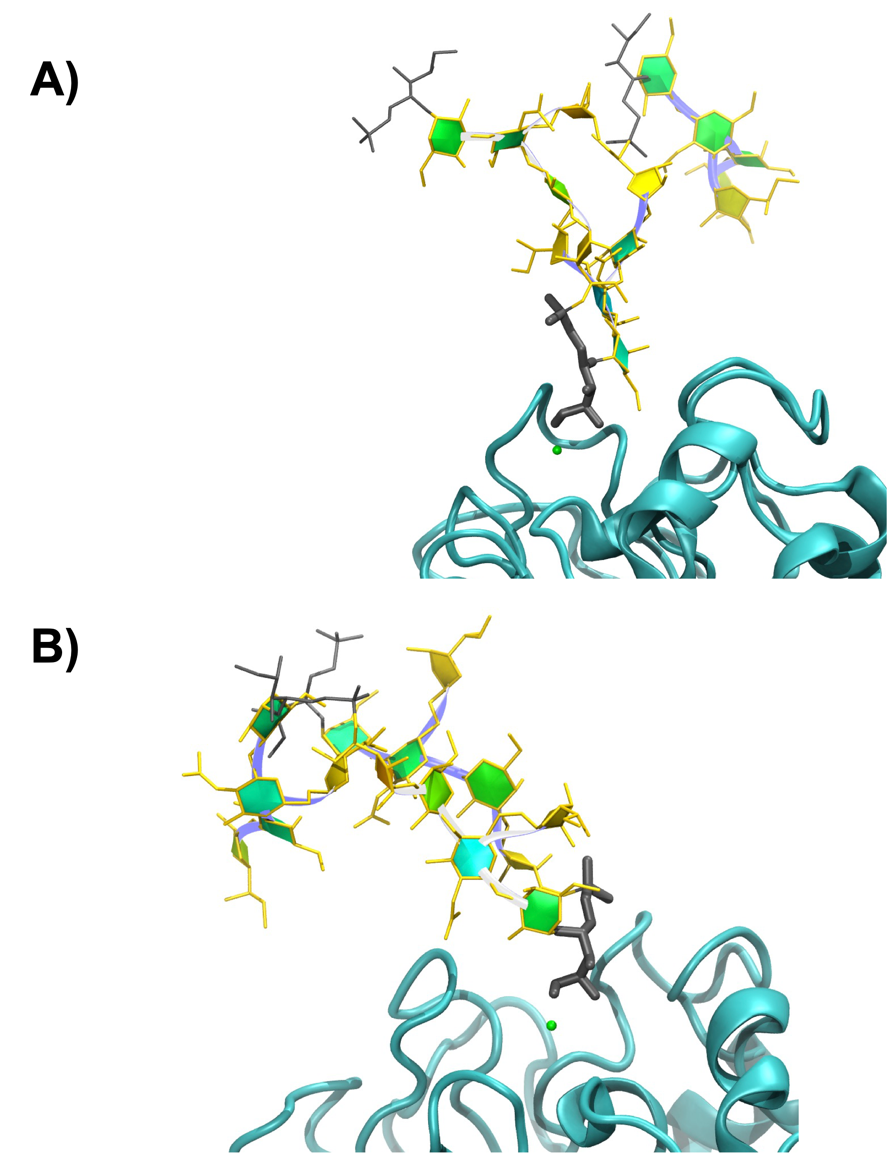


**Supplementary Figure 4.** Alignment of human intelectin-1 bound with allyl-$\beta$-galactofuranose (hIntL-1) with *S. pneumoniae* serogroup 10 CPS Rib-ol-5P ETD moieties. Alignment of 3 RU CPSs RU 42Rib-ol-5P ETD with hIntL-1 binding site for: **(A)** Pn10B, **(B)** Pn10F. Adequate fitting of Rib-ol-5P in the hIntL-1 binding site was achieved by aligning C6, O6, and C3 of the allyl-$\beta$-galactofuranose with O1, C1, and C4 of the Rib-Ol-5P molecule. While we were able to fit the ETD moiety in the hIntL-1 binding site, the stereochemistry of the Rib-ol-5P ETD is opposite to that of molecules expected to bind hIntL-1 and as such further studies are required to establish binding ability of the Rib-ol-5P ETD with hIntL-1 (Isabella, 2021; Kiessling, 2018).

**1.2 References**

Isabella, C. R. (2021). *Carbohydrate and bacterial binding specificity of human intelectin-1* <http://dspace.mit.edu/handle/1721.1/7582>

Kiessling, L. L. (2018). Chemistry-driven glycoscience. *Bioorganic & Medicinal Chemistry, 26*(19), 5229-5238. <https://doi.org/10.1016/j.bmc.2018.09.024>

Richardson, N. I., Kuttel, M. M., Michael, F. S., Cairns, C., Cox, A. D., & Ravenscroft, N. (2021). Cross-reactivity of Haemophilus influenzae type a and b polysaccharides: molecular modeling and conjugate immunogenicity studies. *Glycoconjugate Journal, 38*, 735-746. <https://doi.org/10.1007/s10719-021-10020-0>

stylefix
